# Targeting notch signaling pathway in esophageal cancer: from molecular insights to therapies

**DOI:** 10.3389/or.2026.1709219

**Published:** 2026-07-08

**Authors:** Sangita Bhattacharyya, Hindole Ghosh, Ameer Hamza, Bernhard Biersack, Gregory N. Gan, Prateek Sharma, Prasad Dandawate

**Affiliations:** 1 Cancer Biology, University of Kansas Medical Center, Kansas City, KS, United States; 2 Pathology and Laboratory Medicine, University of Kansas Medical Center, Kansas City, KS, United States; 3 Organic Chemistry Laboratory, University of Bayreuth, Bayreuth, Germany; 4 Radiation Oncology, University of Kansas Medical Center, Kansas City, KS, United States; 5 Gastroenterology and Hepatology, Kansas City Veterans Administration Hospital, Kansas City, MO, United States

**Keywords:** esophageal adenocarcinoma, esophageal cancer, esophagus, inhibitors, notch signaling

## Abstract

Esophageal cancer (EC) is a major digestive cancer with a poor 5-year survival rate of less than 20%. EC incidence is projected to increase by 35% in the US by 2025. The two subtypes of EC with distinct histology, esophageal squamous cell carcinoma (ESCC) and esophageal adenocarcinoma (EAC), exhibit deregulation of various oncogenic signaling pathways, distinct pathological features at diagnosis, and distinct mutation profiles. Hence, these cancers require different treatment approaches for their clinical management. ESCC is more prevalent globally, while EAC is prevalent in Western countries, including Europe, North America, and Australia. Notably, two-thirds of US EC cases are EAC. Drug resistance, faster progression, and recurrence are significant problems of EC. Hence, there is a need to identify novel targets in EC progression. Cancer stem cells (CSCs) are a small population of cells in tumors that can self-renew and are responsible for EMT, drug resistance, and recurrence in EC. These serve as attractive targets for drug development and EC treatment. CSCs express surface markers and use the notch, Hedgehog, Hippo, and Wnt-β-catenin pathways to regulate their growth and survival. We are also including a mutation and expression analysis of PANCAN and EC patients for genes/proteins in the notch signaling pathway, using the cBioPortal and TCGA databases. EC was ranked second among cancers with mutations in the notch pathway. Here, we outline the role of notch signaling in EC biology and its inhibitors for EC treatment.

## 1 Introduction

Esophageal cancer (EC) ranks as the seventh most common cancer worldwide in terms of incidence (604100 new cases) and the sixth leading cause of cancer-related deaths (544076 deaths) (1). EC incidence is rising rapidly, with an abysmal 5-year survival of less than 20% (2). EC is the seventh leading cause of cancer-associated fatalities in the US [estimated new cases: 22,370 and estimated deaths: 16,130 in 2024 (3)] and is projected to increase by 35% by 2025 in the US (4). The biology of EC is relatively poorly understood, and it typically presents with aggressive symptoms at the time of diagnosis (5). Hence, it is among the most difficult cancers to treat or cure. Two histologic types of EC are reported: squamous cell carcinoma [ESCC] and adenocarcinoma [EAC]. Geographically, ESCC is most common worldwide, whereas EAC is predominant in the United States (6).

ESCC accounts for ∼70% of the total EC cases worldwide. ESCC arises from chronic chemical and physical injury to the esophageal mucosa. Tobacco and alcohol use are major risk factors, along with certain high-risk geographic regions containing carcinogens, including polycyclic hydrocarbons, nitrosamines, and acetaldehyde (7–9). Tobacco smoking increases ESCC risk by ∼ 5-9-fold (10,11). Moreover, smoking and alcohol consumption together increase the ESCC risk by ∼3-fold (12). Micronutrient deficiency, resulting from low fruit intake, also increases the risk of ESCC. These risk factors are associated with low socioeconomic status, and countries with weaker economies often have a higher prevalence of ESCC (13). Inherited genetic variants also increase the risk of ESCC. For example, Tylosis, caused by an RHBDF2 mutation, raises risk to ∼90%. Mutations at loci 10q23 (PLCE1), 5q31.2 (TMEM173), 17p13.1 (ATP1B2), and 6p21.32 (HLA class II) are associated with high ESCC risk in the Chinese population (14–16). ESCC progresses from basal cell hyperplasia and dysplasia to carcinoma *in situ*. Multiple studies have reported the progression from esophageal dysplasia to ESCC, suggesting that mutations in TP53, MLL2, and NFE2L2 are involved, while amplifications of SOX2/TP63 and FGFR1 are frequently observed in ESCC cases (17–20). TCGA database showed dysregulation of *EGFR, PIK3CA, FGFR1, TP53, CDKN2A, CCND1, CDK6, MYC, SOX2, KDM6A* and *KMT2D* in ESCC tissues (17–20).

EAC primarily arises from the Barrett’s mucosal layer in the distal esophagus. Gastroesophageal reflux (GERD), Barrett’s esophagus (BE), and obesity are important risk factors for EAC. GERD, hydrochloric acid, and bile acids are major contributors to EAC risk (21,22). Obesity can induce reflux through increased intra-abdominal pressure and is a risk factor for BE and EAC (21,23–26). Other factors include gender (males are at higher risk) and a diet high in red meat and low in fruits and vegetables, which increases the risk of EAC (21,27–30). Typically, EAC develops from metaplasia to dysplasia to a tumor sequence. BE patients are at a 30–125 times higher risk of EAC (31). Acid reflux or bile acids usually damage the esophageal mucosal layer through reactive oxygen species or nitric acid, leading to DNA damage that progresses to EAC. BE preneoplastic tissues are also characterized by frequent genetic alterations that increase the risk of EAC, particularly involving the tumor suppressor genes TP53 and CDKN2 (32,33). Deregulation of p53 signaling plays a vital role in the progression from BE to EAC. EAC has a higher mutation burden than other cancers, including mutations in TP53, ARID1A, and CDK2NA. Copy number alterations, including amplifications and deletions, are observed in genes such as KRAS, EGFR, FGFR2, ERBB2, CDK6, CCND1, MYC, and GATA4, among others. Human papillomavirus (HPV), particularly high-risk types such as HPV-16 and HPV-18, has been detected in ESCC, especially in Africa and China (34–36). However, HPV-16, but not HPV-18, has been found to be associated with ESCC risk. Its role in ESCC remains controversial (37) and may vary by geographic factors (38). Syrjanen and group have summarized that HPV prevalence in EC ranges from 0% to 78% (mean prevalence of 29%) (38). The relationship between HPV and EAC is unclear, as few studies have not detected low or no HPV in EAC (39–41), while some studies have reported an association between HPV16/18 with BE and EAC (42–44). Further research is necessary to clarify the connection between HPV infection and EC risk.

The current clinical EC treatment (45–48) strategy includes surgery (esophagectomy), radiation therapy, chemotherapy, combination therapy, laser therapy, electrocoagulation, and immunotherapy. Chemotherapy, with or without radiation, was administered with drugs such as carboplatin and paclitaxel, oxaliplatin and 5-fluorouracil (5-FU), capecitabine, cisplatin and 5-FU, cisplatin and irinotecan, or paclitaxel and 5-FU or capecitabine. Targeted therapies include trastuzumab, Ramucirumab, Entrectinib, and Larotrectinib. Immunotherapy includes PD-1 inhibitors such as pembrolizumab and nivolumab, as well as the CTLA-4 inhibitor Ipilimumab. Immunotherapy was used for stage II and III patients who are not eligible for surgery and for stage IV EC. According to the NCCN 2024 guidelines (49), the preferred treatment for stage 0 esophageal cancer, characterized by a tumor confined to the mucosa, is endoscopic mucosal resection (EMR). This method is optimal when the lesion remains within the superficial layers and has not penetrated deeper tissues. In scenarios where the tumor is not fully excised or exhibits undesirable features, alternative treatments include submucosal dissection, ablation, or esophagectomy. For stage 1 esophageal cancer, early invasive mucosal endoscopic therapy takes precedence. If there is evidence of submucosal invasion, esophagectomy becomes the primary treatment modality. When the patient presents with low surgical risk or is not a suitable candidate for surgery, endoscopic resection remains the preferred approach. In stage 3 disease, characterized by advanced tumors, the treatment protocol often involves a combined approach of chemotherapy and radiation administered postoperatively. The regimen typically includes Carboplatin and Paclitaxel. If surgical intervention is not feasible, patients may receive definitive chemoradiation. This method is particularly crucial for managing locally advanced disease, notably distal EAC and select squamous cell carcinomas. Standard practice at this stage includes neoadjuvant chemoradiation, in which Carboplatin and Paclitaxel are administered with a total of 41.4 Gy of radiation over 23 treatments spanning 5 weeks. Definitive chemoradiation is employed under several conditions: when surgery is impracticable due to the tumor’s location, when complete surgical removal is unattainable, or when the patient opts against surgical intervention. This treatment remains essential for achieving local control and improving survival outcomes in advanced esophageal cancer cases.

Although several chemotherapeutic treatments are available, the survival outcome in EC patients is abysmal. One reason might be that the treatment regimens for EAC and ESCC are similar, despite exhibiting different pathological and genetic features. Drug resistance, metastasis, and cancer recurrence remain significant problems for EC patients. Therefore, it is necessary to find new pathways and drugs to enhance overall survival in EC patients. Because treatment resistance and locoregional recurrence remain significant problems in EC patients, this suggests that a potentially resistant cell population may reside within the original tumor. Cancer stem cells (CSCs) are a potential culprit because these cells within tumors can self-renew (50), induce drug resistance (51,52), and initiate tumor progression in EC (53,54), making them a promising target for improving cancer control outcomes. These CSCs use various pathways for their growth and can differentiate into other CSCs or other cell types. Several pathways, including notch (55), Wnt/β-catenin (56), Hedgehog (57), and Hippo (58) signaling, have been associated with the propagation and maintenance of CSC, and these pathways are reportedly activated in EAC and ESCC, thereby promoting tumor progression. Currently available therapies do not target CSCs, while several natural products have been shown to eliminate CSCs and inhibit tumor progression (59–62). Targeting these self-renewal CSC pathways by natural or synthetic compounds (63–66) may render them more susceptible to our existing anti-cancer therapies and prevent them from repopulating the tumor bed following curative therapy. Among all self-renewal pathways, the notch pathway showed the greatest promise in both EAC and ESCC. This review aims to summarize the current understanding of the notch pathway and potential inhibitors for EC application.

## 2 Cancer stem cells markers in EC

EC CSCs contribute to tumor initiation, growth, metastasis, chemotherapy resistance and recurrence (67–69). Multiple surface and intracellular markers have been identified on EC CSCs, including CD44, CD90, MUSASHI1, CD271, MAML1, and TWIST1. Moreover, other markers, including ALDH1, OCT3, 4, ABCG2, SOX2, SALL4, BMI1, NANOG and CD133, were reported to be linked with CSCs enrichment in ESCC (68–70). Interestingly, side population, a subset of cells identified by their capacity to efficiently exclude fluorescent dyes, like Hoechst 33342, due to the high expression of ATP-binding cassette (ABC) transporters and often enriched in stem cells and CSCs (71), was used to isolate EC CSCs (72–74) in multiple cell lines, including ESCC (OE21 cells), and EAC (OE19, OE33, and LN1590 cells), which are resistant to drugs (74) such as 5-FU (53) and radiation (75) because of the increased levels of OCT3, 4, β-catenin, and integrin-β1 (76). In addition, Wnt10A upregulation increased self-renewal and the proportion of ESCC CSCs (77). A recent meta-analysis of EC CSC markers revealed that high expression of CD34, CD133, and Nucleostemin was associated with a poor prognosis in EC patients (78). In addition, several other markers have been reported in ESCC, including ICAM1, ALDH1, POU4F1, podophyllin, BMI-1, SALL4, GLI-1, EPCAM, and PIWIL1. ITGA7, RSK4 and LEF1 have been associated with ESCC CSCs and summarized recently (79,80).

## 3 CSC signaling pathways: focusing on notch signaling

CSCs are attracting significant attention, and extensive research has examined the processes that regulate CSC self-renewal, proliferation, and differentiation. CSCs are maintained and regulated by various signaling mechanisms, including the WNT/β-catenin, TGF-β/Smad, Notch, Hedgehog, Hippo, JAK/STAT3, and PI3K/AKT/c-MYC pathways, which are controlled by a regulatory network (80). Unlike normal stem cells, these pathways are heavily mutated and dysregulated in CSCs, including ECs (79,80). These pathways are crucial for CSC regulation and are deregulated in EC. In the following section, we focus on the role of the notch signaling pathway in EC.

### 3.1 Overview of notch signaling pathway

Notch proteins are classified as type I transmembrane proteins that were first identified in *Drosophila melanogaster* (81). Following this, the notch gene was cloned (82), sequenced (83,84), and the notch protein was shown to contain epidermal growth factor (EGF) like repeats (85). The detailed history of notch receptor discovery and progression was recently summarized by Zhou and the group (86). The pathway plays roles in various cancer processes (87), including maintaining stem cells (88), supporting tumor angiogenesis (89), invasion, and metastasis (90). The notch pathway is essential for the development and maintenance of stem cells (60,66). The abnormal notch activation is shown to be linked with cancer progression, including EC (91).

### 3.2 Structure of notch receptors and ligands

**FIGURE 1:**
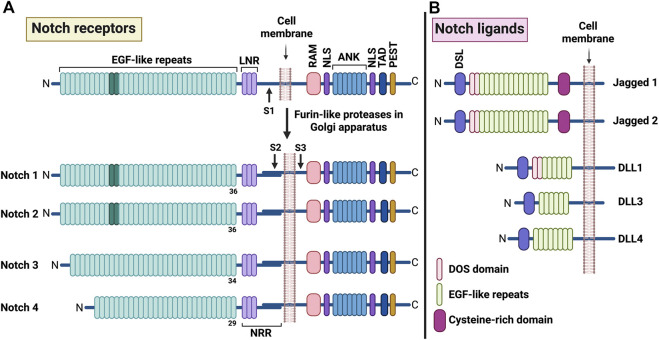
Schematic illustration of the Notch receptors and ligands. **(A)** The figure illustrates the structural organization of Notch receptors, highlighting various domains and sequences: the signal peptide (SP), EGF-like repeats, LIN-12/Notch repeats (LNRs), the RAM domain [which interacts with the Recombination Signal Binding Protein for Immunoglobulin Kappa J Region (RBP-jk)/C Promoter Binding Factor (CBF1)], ankyrin (ANK) repeats, the transcriptional activation domain (TAD), nuclear localization signal (NLS), and the PEST sequence, which is rich in proline (Pro), glutamic acid (Glu), serine (Ser), and threonine (Thr). **(B)** Domain organization of DSL-family (DLL1,3-4) and Serrate family (Jagged 1-2) Notch ligands. The figure highlights the different domains of Notch ligands, including DSL (Delta/Serrate/Lag2), EGF-like repeats, and CRD (cysteine-rich domain). Adapted from (251,252)

Notch signaling enables cell communication. One cell releases ligand (Jagged 1-2, Delta-like ligands 1, 3, 4), which bind to notch receptors (Notch 1-4) on neighboring cells. This causes two cleavages, releasing NICD into the nucleus, where it binds MAML and CSL coactivators to activate target genes (92,93). The notch receptor is a single-pass transmembrane protein with extracellular, transmembrane, and intracellular domains (Figure 1A). The extracellular domain contains the EGF-like repeats 29-36, which are involved in ligand binding (94,95). Additionally, a negative regulatory region (NRR) is crucial for preventing receptor activation in the absence of ligands by blocking S2-cleavage (93). The transmembrane domain (TMD) separates the extracellular part from the intracellular part. The intracellular domain comprises the RBPJ association module (RAM) domain, one nuclear localization signal (NLS), seven ANK (Ankyrin) repeats, two NLSs, and a PEST motif at the C-terminus, which is rich in proline, glutamic acid, serine, and threonine. The ANK repeats recruit nuclear proteins, while the RAM domain recognizes and binds to the transcription factor CBP (CBF-1, Suppressor of Hairless, Lag-2) or RBPJ in mammals (96), and is vital for activating notch-induced transcription in the nucleus. Finally, NLS facilitates NICD transport into the nucleus, while the PEST domain serves as a site for polyubiquitination signaling. This process leads to proteasomal degradation of notch and helps regulate its stability (93,97). Mammals possess three Delta-like (DLL) proteins: DLL1, DLL3, and DLL4, and two Serrate homologs, named Jagged-1 and Jagged-2 (JAG1-2) (Figure 1B). Delta and Serrate ligands possess distinct modular domain structures that are crucial for notch signaling. Starting with an N-terminal MNNL domain, these ligands possess a DSL domain that is crucial for receptor binding. In mammals, ligands such as JAG1, JAG2, and DLL1 also contain two EGF-like repeats, known as the DOS domain, which enhances ligand activity, followed by a cytoplasmic tail at the C-terminal end. Serrate ligands differ from Delta ligands by having a greater number of EGF repeat domains and the presence of a cysteine-rich domain homologous to the von Willebrand Factor C (vWFC) domain, which influences ligand stability and receptor interaction (98,99).

### 3.3 Notch signaling pathway

In signal-receiving cells, notch receptors are synthesized as 300 kDa precursor proteins by ribosomes associated with the endoplasmic reticulum (ER). These precursor proteins undergo initial folding and modifications in the ER before being transported to the Golgi apparatus. Within the Golgi apparatus, they are further processed and cleaved to generate the mature, functional receptor (100). In the Golgi apparatus, furin-like proteases cleave notch receptors at Site 1 (S1), which results in a non-covalent heterodimer consisting of a 180 kDa notch extracellular domain (NECD) and a 120 kDa transmembrane intracellular domain (NTD). This precise cleavage is crucial for the receptor’s functional maturation, enabling it to participate effectively in cellular signaling processes (101). The N-terminal part of notch receptors is O-glycosylated by glycosyltransferase enzymes, such as those in the Fringe family, prior to transport to the cell membrane (102).

**FIGURE 2:**
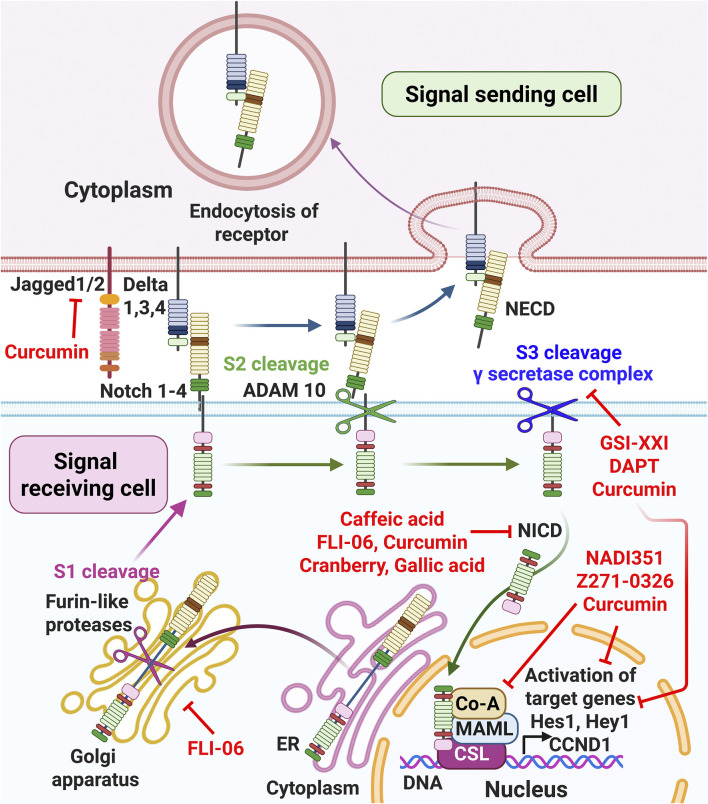
Schematic illustration of the Notch signaling pathway, its regulatory mechanisms, and its inhibitors. This diagram provides an overview of the Notch signaling pathway, emphasizing the steps from receptor production to gene transcription. First, Notch receptors are synthesized and undergo protein folding and glycosylation in the endoplasmic reticulum (ER). They are then cleaved by furin-like proteases at the S1 site in the Golgi apparatus, producing mature receptors that are transported to the cell membrane as heterodimers. Signal-sending cells generate ligands such as Jagged 1-2 and Delta-like (DLL) 1, 3, 4, which bind to Notch receptors (1-4) on target cells. The presence of these ligands and receptors on the cell surface is regulated by endocytosis and membrane trafficking. Ligand endocytosis can generate mechanical forces that induce conformational changes in the Notch receptor, exposing the S2 cleavage site. This enables ADAM proteases to produce the membrane-bound Notch extracellular truncation fragment (NEXT). Next, γ-Secretase cleaves NEXT at the S3 site, releasing the Notch intracellular domain (NICD), which then translocates to the nucleus. Inside the nucleus, NICD binds with RBPJ, Mastermind-like (MAML), and other proteins to form a transcription complex. This complex activates the transcription of target genes, such as HES and HEY, by recruiting coactivators through MAML’s interaction with the NICD/RBPJ interface. This figure illustrates the sequential steps of Notch signaling, including key regulatory points and potential therapeutic targets. Dll1/3/4, Delta-like ligand 1/3/4; ADAM, a disintegrin and metalloproteinase; S1, Site 1; S2, Site 2; S3, Site 3; NEXT, Notch extracellular truncation; NICD, Notch intracellular domain; NTC, Notch transcriptional activation complex; MAML, mastermind-like; CSL, Recombination signal binding protein for immunoglobulin kappa J region (RBPJ), HES, hairy and enhancer of split; HEY, hairy and enhancer of split related with YRPW motif; CCND1, Cyclin D1. Inhibitors are shown in red, and the red line indicates the target for inhibition. Adapted from (93)

In the Canonical or ligand-mediated notch signaling pathway, notch receptors on receiving cells are activated upon binding to notch ligands on adjacent sending cells, triggering the intracellular signaling pathway (103). This binding activates two further proteolytic cleavages; the ADAM-family of proteases mediates the cleavage at site 2 (S-2) (104), producing a transmembrane domain and an intracellular domain (Notch extracellular truncation (NEXT) (105) which is further get cleaved at site 3 (S-3) by γ-secretase complex catalyzes the third cleavage, which comprises PRESENILIN-1, NICASTRIN, APH1, and PEN2, to discharge an intracellular domain of notch receptor (NICD) (106). NICD translocates into the nucleus after release, driven by nuclear localization signals (NLS) recognized by importins α3, α4, and α7, thereby facilitating its entry into the nucleus (107). NICD binds with the DNA-binding factor RBPJ and transcriptional coactivators from the Mastermind-like (MAML) family in the nucleus (108). This complex subsequently binds to target gene promoters, initiating transcription of critical genes (109), including *HES1*, *CCND1*, *MYC*, and *HEY1* [Figure 2, adapted from (110,111)]. This signaling is referred to as “Canonical notch signaling”. In the absence of notch signaling or NICD protein, CSL binds to corepressor factors such as NCoR/SMRT, SHARP (SMRT and HDAC-associated repressor protein), MINT (Msx2-interacting Nuclear Target), HDAC (histone deacetylase), SKIP (Ski-interacting protein), CIR (CBF1-interacting co-repressor) (112–114), L3MBTL3, and the histone demethylase KDM1A (115), among others, and thereby represses the notch signaling.

Non-canonical notch signaling occurs in the absence of DSL ligands or CSL factors, in which the notch receptor regulates aspects such as cell survival, differentiation, and metabolism. Unlike the canonical pathway, which directly triggers transcription, non-canonical pathways commonly engage the PI3K/AKT/Cdc42, Wnt/β-catenin, or NF-κB pathways. There are three different mechanisms. First, it interacts with noncanonical partners to influence processes in the absence of ligands or transcription factors. This includes NICD interacting with non-canonical proteins such as transcription factors like SMAD3 (116), HIF-1α (117), or YY1 (118), which either activate or repress notch target genes. Second, it activates RBPJ-independent pathways in the absence of the usual coactivator, where NICD interacts with various signaling molecules, including PINK1 (69), IKKα/IKKβ (119), GLI2 (120), and NUR77 (121), to modulate their respective pathways. Third, it crosstalks with pathways such as Wnt or NF-κB, integrating multiple networks to regulate cellular functions (122). This mechanism encompasses ligand-independent or non-canonical ligand-dependent activation of notch signaling. For instance, NUMB promotes the breakdown of the notch–β-catenin complex (123); TCR-driven cleavage triggers activation of notch (124); and NICD-driven activation of target genes occurs independently of any requirement for notch ligand. Additionally, various proteins can activate notch signaling. These include membrane-tethered proteins such as DLK1, GPI-anchored proteins such as CNTN1, secreted proteins such as CCN3, and non-canonical ligands such as DAG and PIP3. Recent summaries have covered canonical *versus* non-canonical notch signaling for further reading (122,125).

Notch signaling frequently interacts with other significant pathways that are crucial for tissue development and maintenance. These pathways are frequently disrupted in cancer. They can influence notch signaling either directly or indirectly and notch itself can also modulate their activity. These pathways include phosphatidylinositol 3-kinase(PI3K)/AKT (126), mammalian target of rapamycin (mTOR) (127), epidermal growth factor receptor (EGFR) (128), human epidermal growth factor receptor (HER) (129) from tyrosine kinase, nuclear factor-κB (NF-κB) (130), Wnt-β-catenin (131,132), Hedgehog (133), GSK3 (134), TGF-β (116) and other signaling pathways and proteins (135), which are summarized by some recent articles (95,136).

### 3.4 Status of notch pathway genes in cancer patients

**FIGURE 3:**
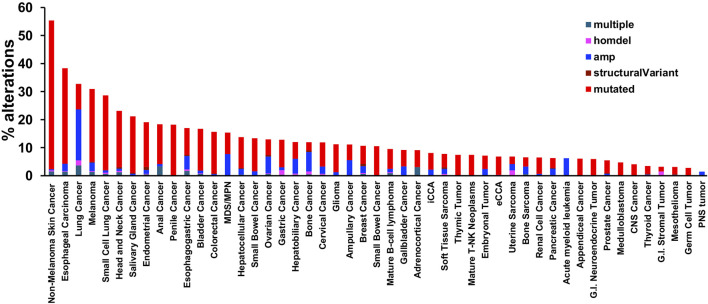
The PANCAN analysis of the combined analysis of alteration in notch receptors 1–4 and notch ligands (Jagged 1-2, DLL1, 3-4). The data is extracted from the c-BioPortal.

Recent comprehensive genome-wide sequencing studies have identified mutations in notch genes across diverse forms of cancer (87). Notably, these mutations vary in location, type, and impact depending on the specific cancer, underscoring the multifaceted roles of notch across oncological contexts (137). Gain-of-function mutations induce persistent and enhanced notch signaling activation, thereby promoting tumor progression. Conversely, loss-of-function mutations reduce notch signaling and slow tumor progression, except in certain cancer types. To understand the mutations in the notch pathway, we analyzed PanCan studies, including MSK-CHORD (138) (MSK, Nature 2024), MSK-IMPACT Clinical Sequencing Cohort (139) (MSK, Nat Med 2017), MSK MetTropism (140) (MSK, Cell 2021), Tumors with TRK fusions (141) (MSK, Clin Cancer Res 2020), China Pan-cancer (142) (OrigiMed, Nature 2022), Metastatic Solid Cancers (143) (UMich, Nature 2017), TMB and Immunotherapy (144) (MSK, Nat Genet 2019), Pan-cancer analysis of whole genomes (145) (ICGC/TCGA, Nature 2020), MSS Mixed Solid Tumors (146) (Broad/Dana-Farber, Nat Genet 2018) using the cBioPortal database and following their standard algorithms. We studied the mutation status of notch receptors (*NOTCH1-4)* and Notch ligands (*JAG1-2, DLL1-4*) in a combined analysis of 76,465 patients. Overall, *NOTCH1-NOTCH3* mutations were present in 4% of patients, whereas *NOTCH2* and *NOTCH4* mutations were present in 3% of patients. Mutations in notch ligands *DLL3* and *JAG1* occurred in 1% of patients, whereas mutations in other ligands were found in less than 1% of patients. Furthermore, the combined mutation frequency of all notch receptors and ligands was highest in non-melanoma skin cancer patients, at 55.38%, followed by EC patients at 38.36% (Figure 3; Table 1). Interestingly, *NOTCH1-4* mutations were most common among non-melanoma patients (44.23%, 29.62%, 21.92%, and 16.54%, respectively), whereas in EC patients, the frequencies of *NOTCH1-4* mutations were 25.9%, 8.52%, 8.03%, and 3.28%, respectively. Regarding notch ligands, the most prevalent mutation was in Jagged1 in esophagogastric cancer, with a frequency of 20.54%. Additionally*, JAGGED 2, DLL1,* and *DLL3* demonstrated the highest alterations in endometrial cancer, whereas *DLL4* mutations occurred in lung cancer with a frequency of 7.89%. The main alteration in NOTCH 1 protein included S2486Rfs*103/S2486Lfs*21/P2485L, *F357del/S/L, X481_*splice/G481S/G481C/G481D, P2514Rfs*4/P2514L/E2515*/P2514A, G2131Afs*117/T2132Hfs*136, A465T, *etc.*, while in the case of NOTCH2, alterations were R2400*/L/Qfs*16, I2304Hfs*9/I2304Lfs*2/P2303S/P2303Hfs*9, S1419Afs*8/P1418L/P1418S/S1419Qfs*22. NOTCH 3 top alterations were G2035Vfs*50/G2035Rfs*60/P2034L/P2034S, A1802Gfs*8/Lfs*23/Gfs*24/S, and P42Lfs*194/C43Lfs*32, etc., while NOTCH4 alterations were G1891Afs*13/2Rfs*24/2Afs*13/1S, E1836K/Q/* and *R385C/H etc.* These mutations were present in EGF-like repeats and ANK domains. JAGGED1 top alteration were D684N, *D466N/X466*_splice, while JAGGED two alterations were *R1185C/L and R996H*, *etc.* It is important to note that these conclusions were based on the information available on the patient population in CBioPortal, and hence, further studies are needed to better understand the burden of notch pathway mutations across various cancer patients. The list of all alterations of notch receptors 1-4 and notch ligands (JAG1-2, DLL1,3-4), including the type of alteration in multiple cancer subtypes, is summarized in Supplementary Table S1.

**TABLE 1:** The PANCAN analysis of the combined analysis of alteration in notch receptors 1-4 and notch ligands (Jagged 1-2, DLL1, 3-4) with cancer subtype and type of alterations. The data is extracted from the C-BioPortal.

Cancer type	Alteration frequency (%)	Alteration type	Alteration Count
Skin cancer, non-melanoma (n = 260)	1.538461538	Multiple	4
	0.384615385	Homdel	1
	0.384615385	Amp	1
	53.07692308	Mutated	138
Melanoma (n = 2,148)	1.35009311	Multiple	29
	0.325884544	Homdel	7
	2.979515829	Amp	64
	0.279329609	StructuralVariant	6
	25.97765363	Mutated	558
Head and neck cancer (n = 983)	1.220752798	Multiple	12
	0.508646999	Homdel	5
	0.813835198	Amp	8
	0.508646999	structuralVariant	5
	20.04069176	Mutated	197
Salivary Gland cancer (n = 283)	0.706713781	Amp	2
	0.35335689	structuralVariant	1
	20.14134276	Mutated	57
Thyroid cancer (n = 726)	0.275482094	Homdel	2
	0.137741047	Amp	1
	0.275482094	structuralVariant	2
	2.754820937	Mutated	20
Esophageal carcinoma (n = 610)	1.475409836	Multiple	9
	0.163934426	Homdel	1
	2.62295082	Amp	16
	34.09836066	Mutated	208
Esophagogastric cancer (n = 1,495)	1.739130435	Multiple	26
	0.535117057	Homdel	8
	4.74916388	Amp	71
	0.401337793	StructuralVariant	6
	9.565217391	Mutated	143
Gastric cancer (n = 866)	0.346420323	Multiple	3
	1.616628176	Amp	14
	1.03926097	StructuralVariant	9
	9.815242494	Mutated	85
Gastrointestinal neuroendocrine tumor (n = 269)	5.94795539	Mutated	16
Small bowel cancer (n = 187)	2.307692308	Amp	3
	12.30769231	Mutated	22
Colorectal cancer (n = 11,494)	0.104402297	Multiple	12
	0.06090134	Homdel	7
	0.400208805	Amp	46
	0.182704019	StructuralVariant	21
	14.88602749	Mutated	1711
Appendiceal cancer (n = 279)	6.093189964	Mutated	17
​	0.833333333	Amp	1
	14.16666667	Mutated	17
Pancreatic cancer (n = 6,409)	0.109221407	Multiple	7
	0.093618349	Homdel	6
	2.293649555	Amp	147
	0.296458106	structuralVariant	19
	3.495085037	Mutated	224
Gastrointestinal stromal tumor (n = 534)	1.498127341	Homdel	8
	1.685393258	Mutated	9
Hepatobiliary cancer (n = 1,632)	0.367647059	Multiple	6
	0.428921569	Homdel	7
	5.208333333	amp	85
	0.06127451	structuralVariant	1
	5.943627451	Mutated	97
Gallbladder carcinoma (n = 240)	3.333333333	Amp	8
	5.833333333	Mutated	14
Extrahepatic cholangiocarcinoma (n = 351)	0.284900285	Homdel	1
	6.552706553	Mutated	23
Liver hepatocellular carcinoma (n = 1,133)	0.353045013	Multiple	4
	2.118270079	Amp	24
	0.26478376	StructuralVariant	3
	11.03265666	Mutated	125
Intrahepatic cholangiocarcinoma (n = 555)	0.18018018	Multiple	1
	1.981981982	Amp	11
	5.945945946	Mutated	33
Ampullary cancer (n = 18)	5.555555556	Amp	1
	5.555555556	Mutated	1
Bladder cancer (n = 1,956)	0.64516129	Multiple	12
	0.204498978	Homdel	4
	0.971370143	Amp	20
	0.051124744	structuralVariant	1
	14.87730061	Mutated	306
Renal cell carcinoma (n = 1,401)	0.071377587	Multiple	1
	0.071377587	Homdel	1
	0.499643112	Amp	7
	5.85296217	Mutated	82
Adrenocortical carcinoma (n = 33)	3.03030303	Multiple	1
	6.060606061	Mutated	2
Bone cancer (n = 201)	0.497512438	Multiple	1
	0.995024876	Homdel	2
	6.965174129	Amp	14
	0.497512438	structuralVariant	1
	2.985074627	Mutated	6
Bone sarcoma (n = 183)	0.546448087	Multiple	1
	2.732240437	Amp	5
	3.278688525	Mutated	6
Soft tissue sarcoma (n = 1,658)	0.301568154	Multiple	5
	0.361881785	Homdel	6
	1.809408926	Amp	30
	0.542822678	structuralVariant	9
	4.76477684	Mutated	79
CNS cancer (n = 49)	4.081632653	Mutated	2
PNS cancer (n = 70)	1.428571429	Amp	1
Glioma (n = 785)	0.127064803	Multiple	1
	0.127064803	Homdel	1
	1.016518424	Amp	8
	9.911054638	Mutated	78
Medulloblastoma (n = 21)	4.761904762	Mutated	1
Acute myeloid leukemia (n = 16)	6.25	Amp	1
Myelodysplastic/Myeloproliferative neoplasms (n = 13)	7.692307692	Amp	1
	7.692307692	Mutated	1
Mature B-cell lymphoma (n = 325)	2.912621359	Multiple	3
	3.883495146	Homdel	1
	0.45045045	Amp	4
	7.657657658	Mutated	23
Mature T and NK neoplasms (n = 27)	7.407407407	Mutated	2
Thymic tumor (n = 54)	7.407407407	Mutated	4
Mesothelioma (n = 351)	0.284900285	Homdel	1
	0.284900285	structuralVariant	1
	2.564102564	Mutated	9
Germ cell tumor (n = 615)	0.162601626	Multiple	1
	2.601626016	Mutated	16
Embryonal tumor (n = 126)	2.380952381	Amp	3
	4.761904762	Mutated	6
Ovarian cancer (n = 1,785)	0.616246499	Multiple	11
	0.224089636	Homdel	4
	5.994397759	Amp	107
	0.504201681	structuralVariant	9
	5.602240896	Mutated	100
Uterine sarcoma (n = 264)	1.893939394	Homdel	5
	2.272727273	Amp	6
	2.651515152	Mutated	7
Endometrial cancer (n = 1,636)	0.488997555	Multiple	8
	0.244498778	Homdel	4
	1.344743276	Amp	22
	1.039119804	structuralVariant	17
	15.95354523	Mutated	261
Cervical cancer (n = 278)	0.35971223	Multiple	1
	0.35971223	Homdel	1
	2.517985612	Amp	7
	8.633093525	Mutated	24
Breast cancer (n = 9,917)	0.614967688	Multiple	59
	0.332761924	Homdel	33
	2.430170414	Amp	241
	0.816779268	structuralVariant	81
	6.443480891	Mutated	639
Prostate cancer (n = 6,297)	0.111164046	Homdel	7
	0.619342544	Amp	39
	0.174686359	structuralVariant	11
	4.589487057	Mutated	289
Lung cancer (n = 55)	3.636363636	Multiple	2
	1.818181818	Homdel	1
	18.18181818	Amp	10
	9.090909091	Mutated	5
Small cell lung cancer (n = 618)	0.647249191	Multiple	4
	0.485436893	Homdel	3
	0.809061489	Amp	5
	26.69902913	Mutated	165
Non-small cell lung cancer (n = 16,612)	0.307006983	Multiple	51
	0.25282928	Homdel	42
	0.51769805	Amp	86
	0.25282928	AtructuralVariant	42
	11.46761377	Mutated	1905
Cancer of unknown primary (n = 419)	0.715990453	Multiple	3
	0.238663484	Homdel	1
	1.431980907	Amp	6
	0.715990453	StructuralVariant	3
	14.55847255	Mutated	61
Penile cancer (n = 11)	18.18181818	Mutated	2

### 3.5 Status of notch pathway genes in EC patients-TCGA analysis

**FIGURE 4:**
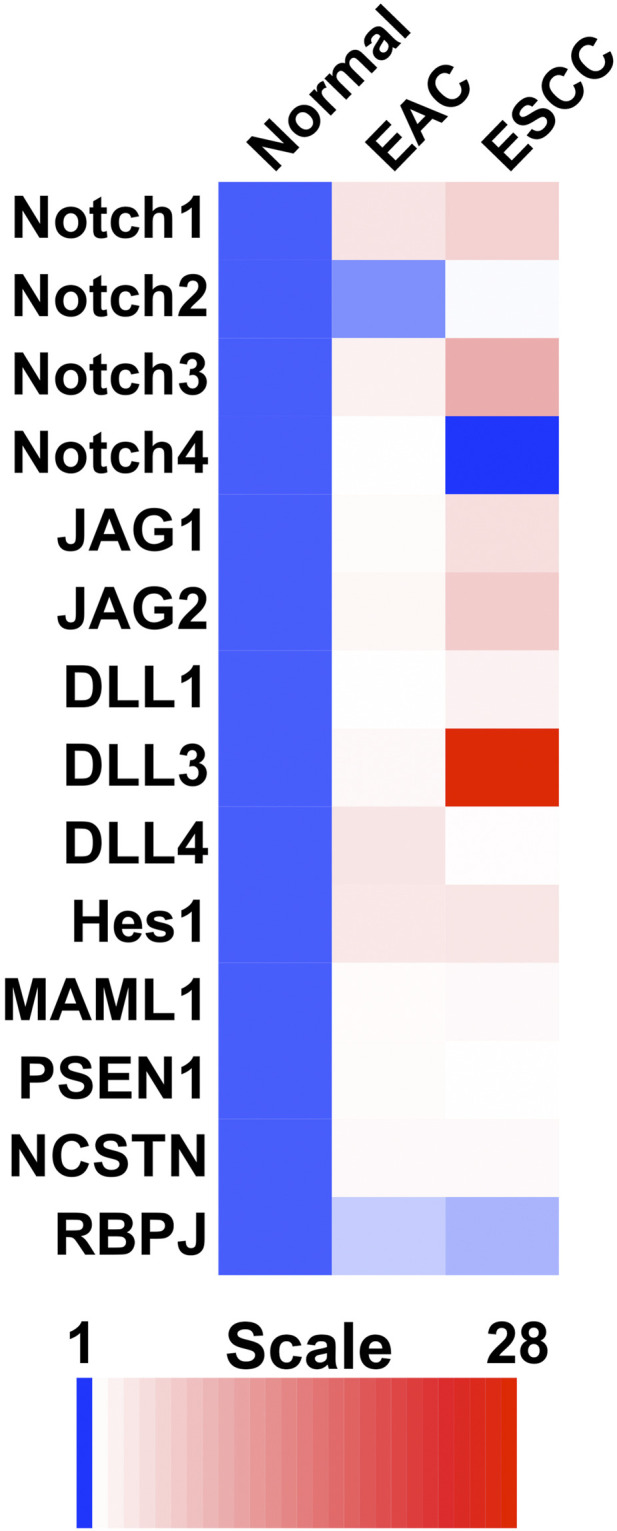
The levels of notch signaling pathway genes in normal, EAC, and ESCC tissues. The values are expressed as fold changes relative to normal tissues, and the data are obtained from the UALCAN browser.

To gain insight into the expression of notch pathway genes in EC, we initially analyzed the TCGA database utilizing the UALCAN browser (https://ualcan.path.uab.edu/analysis.html) (March 2024). Our investigation focused on median notch signaling gene expression levels (transcripts per million) in esophageal cancer (EC) tissues and compared them with those in normal esophageal tissues from the EC cohort. The p-values were obtained by the standard algorithms of the UALCAN. We found that *NOTCH1, JAGGED1, JAGGED2, DLL1, HES-1, MAML1, PRESENILIN1, and NICASTRIN* were significantly overexpressed (p < 0.05, Table 2) in both EAC and ESCC tissue samples. Notably, NOTCH3 and *DLL3* were selectively overexpressed in ESCC tissues. Furthermore, *NOTCH 1–3, JAGGED1, JAGGED2, DLL1*, and *HES1* were expressed at significantly higher levels in ESCC tissues than in EAC tissues, while *DLL4* and *PRESENILIN1* were markedly higher in EAC compared to ESCC tissues (Figure 4; Table 2). This suggests that the notch pathway is upregulated in EC. In our investigation of mutations in notch pathway genes in EC tissues, we used cBioPortal for analysis. We identified mutations in *NOTCH1* (8% each), *NOTCH2* (4% and 3%, respectively), *NOTCH3* (5% and 1.9%, respectively), and *NOTCH4* (4% each) in both EAC and ESCC tissues. Notably, *HES1* exhibited the highest mutation rate (10%), but this was observed exclusively in EAC tissues. All notch pathway genes under examination showed mutation rates ranging from 0.9% to 10% in EAC tissues; however, no mutations were reported in *HES1, PRESENILIN1, NICASTRIN, JAGGED1, JAGGED2, DLL3, or MAML1* within ESCC tissues (Table 3). Overall, we found that the notch signaling pathway is dysregulated in both EAC and ESCC patients, and further studies are needed to confirm TCGA findings by validating them in patient tissues *via* immunohistochemistry or other biochemical techniques to detect protein levels and correlating them with patient demographics, including disease stage, gender, race, chemotherapy status, *etc.*

**TABLE 2:** The levels of notch signaling pathway genes in normal, EAC, and ESCC tissues. The values are expressed in transcripts per million, and the data is extracted from the UALCAN browser.

Transcripts per million				
Gene	Normal	EAC	ESCC	EAC vs ESCC
Notch1	6.055	25.852*	37.435**	**
Notch2	22.333	25.845	33.186	**
Notch3	17.306	51.885	170.863**	**
Notch4	7.272	11.983	6.291	**
JAG1	23.952	46.477**	116.509**	**
JAG2	5.595	13.525*	37.304**	**
DLL1	4.384	7.39**	12.787**	**
DLL3	0.012	0.027	0.331**	ns
DLL4	2.04	8.553	3.548	**
Hes1	28.406	110.559**	116.123**	**
MAML1	13.444	25.429*	29.013*	ns
PSEN1	19.338	38.007**	31.555**	**
NCSTN	51.447	111.714**	112.853**	ns
RBPJ	27.008	36.471	34.48	ns

*p<0.05, **p<0.01, ns, not significant.

red, ESCC>EAC; blue, EAC>ESCC.

**TABLE 3:** The list of mutations in the notch signaling pathway genes in EAC and ESCC tissues. The % of mutation incidence is calculated, and the data is extracted from the c-bioportal.

Esophageal adenocarcinoma (% alterations)														
Notch 1 (8%)	Notch 2 (4%)	Notch 3 (5%)	Notch 4 (4%)	HES1 (10%)	PSEN1 (0.9%)	PSEN2 (1.8%)	NCSTN (2.4%)	RBPJ (1.2%)	JAG1 (1.8%)	JAG2 (4%)	DLL1 (1.2%)	DLL3 (2.1%)	DLL4 (1.2%)	MAML1 (1.5%)
R365C E2012* A86Cfs*57 E758Ifs*50 P157Afs*99 X367_splice D1520Afs*59 E1294* N304Mfs*327 N927Qfs*17 X1723_splice R1783W R2070Q R2179Q C1528R G766A A1696V S673T E488K C1122S	X292_ splice A3V G313D R1718C S1020F E990K	R113* R883* X651_splice W1003* C1250Wfs*10 R1546H R1669C Q1427H P359T A880T S396Y C1417S D1481Y PTP4A1-NOTCH3 Fusion	A1560V C326F G704R G1109C V600I D1404N D1356E A1729V P686T A405D	S11L V85A	M16I M292I	R29H R110H T301M	V96A V354M	H52L C68Lfs*27 P99L	V769I S32* E711G	A1225V R195C V462I A1051V S391W T1066A	K535R E181K R570W	LRFN1-DLL3 Fusion X290_splice	C399G G452S	G762C R783Q
A465T A465T X1978_splice D680Rfs*2 X2060_splice Q1763* K2054Rfs*213 N151Rfs*104 E1148* Y662* C1514Wfs*8 P460L C456Y E424K D374Y C74F G484V E948K E1265K	X1668_splice C471F N627S Y1134C C611Y F360C C131R	S497L D1936G	​	​	​	​	​	F146L	​	​	L643F	​	E566G R446H R420L D635N R624Gfs*15	​

### 3.6 Notch signaling in the esophagus

Notch receptors 1-3 are expressed at higher levels in the normal human esophagus and are important for maintaining the integrity of the squamous epithelial layer (147,148). NOTCH1 regulates squamous differentiation in the normal esophagus (149). Interestingly, the epidermis still formed normally in the mice even after deletion of notch receptors 1-3, but a deficiency in barrier function and squamous hyperplasia was noted (149). Moreover, notch signaling controls the differentiation of esophageal squamous epithelium through an *HES-1*-dependent mechanism (150,151) and regulates essential regulator genes involved in squamous differentiation, including *p63* (152), *NRF2* (153,154), *IRF6* (155), and *HPV8 E6* (156). The notch pathway is crucial for mouse esophageal epithelial development (157), and genetic deficiency of notch pathway proteins such as RBPJ, JAGGED1, and JAGGED2 has been shown to impair esophageal squamous differentiation in mice. The notch pathway is also critical in generating esophageal progenitor cells from stem cells (148). Deletion of NOTCH1 reduced tumor growth, mirroring the effects of anti-NOTCH1 antibody treatment. Tumors lacking NOTCH1 demonstrated decreased proliferation. The study concludes that mutations in NOTCH1 within normal epithelium confer a selective benefit, as wild-type NOTCH1 promotes tumor expansion and growth. Inhibiting NOTCH1 could have therapeutic potential for preventing ESCC (158). These reports showed that the notch pathway is crucial for normal esophageal development and maintenance.

### 3.7 Notch pathway in the normal esophagus and its association with various diseases

Even after the significance of notch signaling in esophageal development was established, mutations were reported in normal esophageal tissues (approximately 66.2% of tissue samples) and in ESCC (15% of tissue samples). Interestingly, the *NOTCH1-3* mutation pattern showed striking similarities between normal and ESCC samples. Martincorena and coworkers (159) recently studied the incidence of somatic mutation clones in the normal esophagus and their association with age. The results showed that 14 genes were frequently mutated in the normal esophagus, with notch receptors 1, 2, and 3 among the top 5 mutated genes. Across nine normal esophagus samples, 2,055 coding mutations were detected in *NOTCH1*; among these, 98% were nonsynonymous, with approximately 120 different *NOTCH1* mutations per square centimeter of the normal esophagus. Moreover, *NOTCH1* mutations were positively correlated with age. In summary, notch signaling is critical in esophageal biology. Whole-genomic and whole-exome sequencing of ESCC tissues at different stages of the disease (Stage 1, n = 51; Stage 3, n = 53) revealed the prevalence of *NOTCH1* mutations in ESCC tissues (160). Molecular analysis of 43 patients with locally advanced and metastatic ESCC revealed *NOTCH1* as one of the commonly mutated genes, with a mutation rate of 26.2%. NOTCH1 mutations were mutually exclusive with PI3KCA and associated with poor overall survival (161). *NOTCH1* mutations are more commonly seen in early-stage tumors, particularly in Stage 1. These mutations are closely linked to well-differentiated tumors and a lower risk of lymph node metastasis. For example, one study found that 18 of 60 Stage 1 tumors had *a NOTCH1* mutation, compared to only 4 of 44 Stage II or Stage III tumors (162). *NOTCH1* mutations are relatively rare, suggesting that they promote clonal expansion but hinder carcinogenesis (Abby, 2023 #161). Recently, a genomic study, coupled with GO pathway analysis, of ESCC cell lines revealed that genes in the *HIPPO, NOTCH1-3, PI3K, RTK-RAS,* and Wnt-β-catenin pathways were mutated in the EC cell lines, including *TE-1, ECA-109, KYSE-30, -150, -180, -450,* and −510cell lines (163). However, some reports suggest a higher prevalence in early-stage disease; further research is needed. Similarly, genomic analysis of 183 ESCC patients revealed that the notch pathway is altered in 38.3% of patients (164). Hence, it is interesting to recognize the role of the notch pathway in esophageal diseases, including gastroesophageal reflux (GERD), Barrett’s esophagus (BE), and EC (ESCC and EAC).

#### 3.7.1 Notch in GERD and BE

GERD refers to the regurgitation of gastric and duodenal fluids into the esophagus, causing heartburn and upper abdominal or chest pain. Acid and bile acid interactions with esophageal squamous cells reduce notch signaling, favoring goblet cell (GC) differentiation (165–167). Treatment with a γ-secretase inhibitor (GSI) converted BE proliferative cells into differentiated GCs while keeping the squamous epithelial layer intact in reflux-induced BE in rats (168). These data suggest an important role for notch inhibition in human BE. Alcohol is another risk factor associated with GERD and BE. Ethanol treatment inhibits the notch pathway by suppressing the activity of the γ-secretase complex (169). Ethanol exposure decreases *PAX9* expression, a downstream target of the notch pathway, *in vitro* in human esophageal epithelial cells (170). Moreover, esophageal *PAX9* deficiency leads to increased cell proliferation and impaired cell differentiation in mice (170). Recent *RBPJ* and *NICD1* ChIP-PCR studies have confirmed that *PAX9* is a target of *NOTCH1* signaling in the squamous cells of the esophagus. This suggests that the *NOTCH-PAX9* signaling axis plays a role in ethanol-induced esophageal injury (171,172). Wang and colleagues demonstrated that notch signaling pathway proteins, including *NOTCH1* and *HES1*, are expressed at lower levels in BE tissues obtained from 36 patients compared to the normal esophagus. Interestingly, MUC2 expression was enhanced upon NOTCH1 inhibition induced by siRNA in Het1A cells. This group also confirmed that hydrochloric acid and deoxycholic acid inhibited *NOTCH1* and *HES1 expression,* leading to increased MUC2 expression in Het1A cells (165). The inhibition of notch signaling in BE was also confirmed by another clinical study of 48 patients undergoing esophagectomy with gastric interposition (166). In another study, the differential roles of bile acid-induced CDX2 expression in esophageal stem cells and reduced notch signaling in BE development were examined. The study showed low *HES1* and high *MUC2* and *ATOH1* expression in intestinal metaplasia with goblet cells in the esophagus. Moreover, forced expression of *CDX2* and bile acid treatment in cells inhibited HES1 and increased MUC2 and ATOH1 expression. In summary, increased *CDX2* expression caused by bile acids promotes intestinal differentiation in esophageal cells by suppressing notch signaling proteins (173). Vega et al. demonstrated that esophageal epithelial trans-differentiation toward BE-like metaplasia, induced by inhibition of notch signaling, was partially associated with upregulation of the KLF4 protein (174). Notch2 was also studied in relation to BE development, specifically in the DCLK1-expressing tuft cells. Notch pathway initiation in *DCLK1-*expressing tuft cells led to a higher incidence of BE in *p-EDL2-DCLK1. N2IC* mice, while Notch2 deletion in *DCLK1-*expressing cells slowed BE progression in *p-EDL2-DCLK1-N2fl* mice. These data suggest that activation of Notch signaling in *DCLK*1 cells contributes to BE development (175). A recent study showed that *P53* mutation increased progenitor expansion and inhibited stem cell differentiation by activating notch, leading to a shift from metaplasia to dysplasia in L2-IL1β mice (176). Overall, notch signaling plays a dual role in BE, acting as an initial promoter of metaplasia while its subsequent overactivation drives progression to EAC. Increased notch signaling reduces goblet cell differentiation and correlates with dysplasia. Conversely, inhibiting notch can induce intestinal transdifferentiation. Hence, further studies are needed to establish the mechanistic role of notch signaling in BE progression.

#### 3.7.2 Notch signaling pathway in ESCC

Notch signaling has been reported to have both tumor-suppressing and tumor-promoting effects in ESCC (Table 4) (177). In the esophagus, notch signaling serves a tumor-suppressive role. Treatment with the chemical carcinogen 4-nitroquinoline 1-oxide (4-NQO), which is known to induce oral and ESCC, induced Notch1 loss in the basal cells of the normal esophageal squamous epithelium, inducing skin SCC but not in the esophagus. Additional studies reported inhibition of NOTCH1 in 4-NQO-induced head and neck and oro-esophageal SCC (178,179). Mice with conditional Notch1 cKO [generated by crossing a strain that expresses Cas9 driven by the human KRT14 promoter with a guide RNA targeting Notch1 exon two under the human U6 promoter (KRT14-Cas9; gRNA/Notch1)], in which Notch1 expression was disrupted specifically in squamous epithelium, developed tumors earlier in response to 4-NQO. The mechanism involved NOTCH1 regulating *TERT* expression, and the authors concluded that a lack of NOTCH1 increased the risk of ESCC tumor development in affected epithelium, partly by accelerating telomere erosion (179). Moreover, similar results were reported with the chemical carcinogen diethyl-nitrosamine, where notch inhibition resulted in increased tumor size and numbers in the esophagus (180). Here, the authors generated mice expressing a dominant-negative MAML1 (DNMAML1), which inhibited NICD transcription and induced DNMAML1 in the basal layers sporadically. When DNM mice were treated with the carcinogen diethyl nitrosamine, lesion area and tumor induction were increased compared to control mice (180). This study also used lineage tracing experiments with conditional DNMAML1. The results showed that inhibition of the notch pathway increased the differentiation of neighboring wild-type progenitor cells and prevented the differentiation of mutant progenitor cells, ultimately replacing the entire epithelial layer (180). In another study, NOTCH3-mediated signaling inhibited the expansion of zinc finger E-box binding (ZEB)-expressing cells, while inhibition of NOTCH3 signaling by dominant-negative Mastermind-like1 (DNMAML1) reduced squamous cell differentiation and notch downstream gene expression in EPC2-T cells. DNMAML1*-*expressing cells exhibited significant upregulation of *ZEB1 and ZEB2*, downregulation of the miR-200 family, increased colony formation, and enhanced tumor progression in nude mice. RNA interference studies have shown a connection between ZEBs and cancer progression and EMT. In the 3D-organotypic model, notch inhibition by *DNMAML1* recapitulated growth and compromised squamous differentiation. Moreover, knockdown of NOTCH3 by shRNA promoted EMT and upregulated ZEB1-2, while impairing squamous differentiation in EPC2-hTERT esophageal cells. This report suggested that NOTCH3 is a key protein that regulates or reduces the expansion of *ZEB*-expressing cells and plays a role in determining cell fate in ESCC (175). Taleb et al. measured HES1 mRNA levels in 50 ESCC tumor samples and their adjacent normal tissues using RT-PCR. The study reported the downregulation of HES1 mRNA and its correlation with invasiveness in ESCC (181). There was also a report of reduced NOTCH1 expression in ESCC patient tissue (182). Deleting JAGGED1 and JAGGED2 in mice disrupted cell balance in the esophageal and forestomach epithelium by impairing notch signaling, reducing squamous epithelial cells, and increasing basal progenitors. It worsened deoxycholic acid–induced injury and SCC in the forestomach. Notably, *JAG1/2* levels are lower in early human ESCC. This study suggested that JAGGED1/2 are crucial for the onset of forestomach SCC (183). Recently, a single-cell transcriptomic analysis revealed that knocking out *TRP53, CDKN2A, and NOTCH1* (PCN) together induces cancer-like features in ESCC by creating cell lineage diversity and high cell plasticity. Furthermore, PCN knockout also creates an immunosuppressive environment characterized by an abundance of exhausted T cells and M2 macrophages, mediated by the CCL2-CCR2 pathway in ESCC initiation (184). Furthermore, the overexpression of the NOTCH3 intracellular domain has been shown to inhibit EMT and to sensitize ESCC cells (TE6 and TE11) to chemotherapy (5-FU), and is proposed as a potential new biomarker for predicting outcomes in ESCC patients clinically (185). Recently, comprehensive tumor genomic profiling and transcriptome sequencing were conducted on samples from the Phase 3 RATIONALE-302 study of Tislelizumab in patients with advanced or metastatic ESCC to elucidate NOTCH1 mutations and patient survival outcomes. NOTCH1 mutation (loss-of-function, indicated by reduced NOTCH1 and its downstream targets HEY2 and HEYL) was identified as a predictive biomarker for longer overall survival (OS) with tislelizumab compared to chemotherapy. Type I IFN/toll-like receptor expression signatures were positively associated with OS benefit, while B-cell and neutrophil signatures predicted unfavorable OS. The presence of NOTCH1 mutation correlated with enrichment of IFN-I signatures and reduced B-cell and neutrophil infiltration. Interestingly, in murine xenograft models using the ESCC cell line HNM007, Notch1 deficiency (shRNA-NOTCH1 KD) was found to promote a more immunologically activated tumor microenvironment, enhancing the efficacy of anti-PD-1 treatment and providing a rationale for combining notch signaling inhibitors with anti-PD-1 therapy to improve antitumor efficacy against ESCC (186).

**TABLE 4:** Key roles of notch signaling in ESCC.

Mechanisms	Description	Key genes/proteins involved
Tumor suppression	• Maintains epithelial homeostasis and squamous differentiation. • Loss or inhibition accelerates tumorigenesis. • Downregulation or mutation impairs differentiation and increases cancer risk. • Notch1 mutations often predict better immunotherapy response and a more active immune microenvironment.	NOTCH1, NOTCH2, NOTCH3, NOTCH4 (receptors); JAG1, JAG2, DLL1, DLL3, DLL4 (ligands); HES1, HEY1 (effectors)
Tumor promotion	• Switches to oncogenic role during progression. • Promotes EMT, stemness, metastasis, and poor prognosis. • EMT is mediated by upregulation of transcription factors and crosstalk with other pathways. • Enhances CSC properties.	NOTCH1, NOTCH3, MAML1 (co-activator); EMT drivers: SNAIL, ZEB1, ZEB2, TWIST1; CD44 (CSC marker); HES1
Drug resistance	• High NOTCH1/NOTCH3 activity and upregulation of MAML1, SOX2, SALL4, NANOG, and HES1 associated with chemoresistance and radioresistance. • Maintains CSC subpopulations (CD44-high), upregulates EMT, and activates survival pathways, diminishing therapy efficacy.	NOTCH1, NOTCH3, MAML1, SOX2, SALL4, NANOG, HES1, CD44
Pathway crosstalk and regulation	• Interacts with TGF-β (promotes EMT), Wnt/β-catenin (radioresistance), EGFR/MSI1 (proliferation), and metabolic/epigenetic regulators (PRMT1, POFUT1, METTL3, LSD1/KDM1A). • Crosstalk amplifies effects on EMT, stemness, and therapy resistance • Modulated by lncRNAs (HAGLROS, SNHG1), inflammation (IL-23), and immune microenvironment.	TGFB1 (TGF-β), CTNNB1 (β-catenin/Wnt), EGFR, MSI1, PRMT1, POFUT1, METTL3, LSD1/KDM1A, HAGLROS, SNHG1, IL-23
Clinical implications	• NOTCH1, NOTCH3, and HES1 mutations or expression levels serve as prognostic and predictive biomarkers • NOTCH pathway activity predicts response to immunotherapy (e.g., anti-PD-1/PD-L1 antibodies like tislelizumab). • Components are potential therapeutic targets (e.g., MAML1 inhibition for CSCs), but therapeutic strategies require careful context-dependent consideration due to dual roles.	NOTCH1, NOTCH3, HES1, MAML1; immunotherapy agents (tislelizumab, anti-PD-1/PD-L1)

Although few reports have examined the tumor-suppressor roles of notch pathway proteins, there are no detailed studies that provide a clear, context-dependent framework (e.g., disease stage, mutation profile, or microenvironment). Numerous studies have reported tumor-promoting effects of notch proteins in ESCC. Notch is believed to become carcinogenic during tumorigenesis, a process that is also contingent on genetic and cellular background. Single-cell RNA sequencing has revealed that ESCC tumors with elevated notch activity are associated with markedly poorer survival outcomes than those with diminished notch activity (187). Interestingly, Kagawa et al (188) studied cell senescence checkpoints and their interactions with the notch pathway, revealing both tumor-suppressing and tumor-promoting effects. The authors used EPC1 and EPC2 (human esophageal keratinocytes) and their derivatives, as well as ESCC cell lines (EN60 and TE11). Ectopic expression of NICD1 conferred oncogenic properties, including cell senescence, G0/G1 arrest, Rb phosphorylation, changes in cell morphology (flat and enlarged), and increased β-galactosidase activity. Furthermore, they discovered that notch-induced senescence relies on CSL-RBJP-mediated transcription and *P16* ^*INK4A*^ *-RB* signaling. siRNA-mediated loss of *P16* ^*INK4A*^ inhibited NICD1-induced senescence; similar effects were observed with HPV E6/E7, which are known to inactivate p53 and Rb. Next, the authors analyzed the effect of tetracycline-inducible NICD1 expression in already transformed ESCC cell lines (EN60 and TE11) and found that NICD1 increased colony formation and tumor xenograft growth in nude mice. These data suggest that notch-mediated cellular senescence contributes to the notch-mediated tumor-promoting effect (188).

Furthermore, NICD1 and epithelial-mesenchymal transition were identified as features of ESCC progression in the 4NQO mouse model. Increased activated NICD1 (Val1744) was reported in dysplastic lesions and was high in ESCC tumors. Moreover, NICD1 expression was detected in invasive ESCC cells exhibiting E-cadherin mislocalization and ZEB upregulation, suggesting Notch1 involvement in EMT in ESCC. Furthermore, Notch1 expression promoted ESCC carcinogenesis and expanded CD44H cells with mesenchymal characteristics. These EMT-promoting effects of Notch were found to be associated with transforming growth factor (TGF)-β, which is present in the tumor microenvironment. TGF-β activated the expression of ZEB1 to inhibit NOTCH3 activity. TGF-β activated the Notch1-associated EMT to increase CD44-high cancer-initiating cells. Furthermore, NOTCH1 expression is observed at the invasive front of ESCC tumors and is associated with a poor prognosis. This study highlighted the relationship between NOTCH1 and NOTCH3 in the initiation of EMT and ESCC (189). Hypoxia-inducible factor-2α (HIF-2) was shown to regulate the migration, proliferation, and EMT of ESCC cells (Eca-109 and KYSE-150*)* through the notch signaling pathway (190). Similarly, Fahim et al. (191) recently demonstrated that TWIST1, another EMT-inducing protein, contributes to the aggressiveness of ESCC by regulating notch signaling genes. The study evaluated the expression and correlation of TWIST1 and notch pathway genes in ESCC and normal esophageal tissues. TWIST1 expression was significantly correlated with HES1/HEY1 expression and linked to poor prognosis in ESCC. Moreover, overexpression of TWIST1 in ESCC cells (KYSE30) activated notch signaling gene expression. These datasets confirm previous findings that EMT and notch signaling are involved in the progression and aggressiveness of ESCC. In addition, silencing MAML1 led to downregulation of TWIST1, whereas ectopic MAML1 expression increased TWIST1 expression at both the mRNA and protein levels in KYSE-30 cells. Expression of mesenchymal genes increased following overexpression of MAML1 and TWIST1. The study found that MAML1 is an upstream regulator, as TWIST1 overexpression did not affect MAML1 levels in KYSE30 cells (192). Interestingly, concomitant expression of MAML1 and TWIST1 was observed in ESCC patients. Overexpression of these proteins has also been associated with metastasis and advanced ESCC disease (Stages III and IV). MAML1’s mean relative expression (MRE) was higher in ESCC patients with lymph node metastasis, while TWIST1 MRE was increased in ESCC patients with tumor invasion into the adventitia (193). In a similar study, MAML1 and homeobox transcription factor MEIS1 mRNA were shown to be negatively correlated in ESCC tissues (194).

Another population of cells in cancer that exhibits an EMT phenotype is CSCs. Both EMT and CSCs are involved in cell senescence (195,196) and drug resistance. Esophageal CSCs are one of the reasons for tumor relapse in ESCC. Moghbeli et al. isolated CD44^+^ ESCC CSCs, which formed tumor xenografts in mice. These CSCs overexpressed markers like NANOG, SOX2, OCT4, and notch target genes HES1, HEY1, MAML1, compared to CD44-negative cells. To study notch signaling’s influence on tumor formation, MAML1 was silenced in CD44-positive cells, resulting in reduced migration, increased 5-fluorouracil (5-FU) resistance, and increased the number of cells in G1 phase, whereas *MAML1* ectopic expression enhanced migration, reduced 5-FU resistance, and increased the number of cells in S phase. This study suggested using MAML1 as a targeted therapy for CD44-positive CSCs in ESCC (197). Similarly, whole-cell small RNA sequencing and transcriptome sequencing revealed that cisplatin-resistant EC cells exhibit enrichment of the EMT and notch signaling (198). RT-PCR analysis of ESCC tumors (n = 50) and adjacent normal tissues showed that the overexpression of ESCC CSC markers SOX2 and SALL4, along with the concomitant activation of notch signaling, was associated with tumor invasion and lymph node metastasis, suggesting crosstalk between *SOX2 and SALL4* and notch signaling in ESCC stemness, invasion, and metastasis (199). Potassium channel tetramerization domain containing 12 (KCTD12) was shown to regulate SALL4, NANOG, SOX2, MAML1, and other stem cell markers in the ESCC cell line KYSE30 (200). Interestingly, the knockdown (RNAi) of SOX15 was shown to inhibit ESCC proliferation and sensitize ESCC cells to paclitaxel, but not cisplatin, by downregulating the notch signaling pathway (201). Zhao et al. found that PRMT1 is linked to histological grade, tumor stage, and poor prognosis in ESCC patients. Moreover, PRMT1 is overexpressed in TICs, promoting stem-like traits, chemotherapy resistance, and tumor growth in Eca109 OV6+ cells in NOD/SCID mice. It regulates notch proteins, including NOTCH1, NOTCH2, JAGGED1, HEY1, and HES1, in TE1 and ECA109 ESCC cell (202). Additionally, glycol-gene *POFUT1* overexpression promoted proliferation and metastasis in ESCC and was linked to a poor prognosis in ESCC patients. This overexpression resulted in increased overall fucosylation and activated the notch signaling pathway, contributing to *POFUT1-*driven pro-migration ESCC (203). Long non-coding RNA HAGLROS controls the proliferation, migration, and apoptosis of ESCC cells (Eca109 and KYSE150 cells) through the HAGLROS-miR-206-Notch3 axis in ESCC cells and KYSE150 cell xenografts in nude mice (204). Ubiquitin-protein ligase E3A (UBE3A) expression was shown to enhance EC progression by activating ZNF185/NOTCH signaling axes in ESCC cells (ECA-109 and TE1) (205).

Inflammatory cells within the tumor microenvironment significantly influence tumor behavior and aggressiveness. The pro-inflammatory cytokine Interleukin 23 (IL-23) exerts pro- or antitumor effects through the IL-23 receptor. IL-23 is significantly expressed in ESCC tumors and colocalized with OCT4A in IL-23 receptor-positive ESCC cell lines. Furthermore, treatment with IL-23 significantly increased the CD133+ cell population and triggered notch signaling in CD133-negative, IL-23R-positive ESCC cells. At the same time, upon IL-23 pretreatment, these cells showed resistance to radiation-induced apoptosis, with higher survival rates than the untreated control group. These radioresistance and dormancy in IL-23-pretreated cells were reversed by small-molecule or siRNA inhibition of the Notch and Wnt pathways. These datasets revealed the role of IL-23-regulated Notch/Wnt signaling in radioresistance in ESCC (206). Another study reported the crosstalk mechanism between the Notch and Wnt pathways in ESCC. Abbaszadegan et al. reported a significant association between epidermal growth factor receptor (EGFR) and Musashi1 (MSI1) and tumor grade in ESCC, suggesting a direct correlation between MSI1 and EGFR in the activation of the Notch/Wnt pathways in ESCC (207). Han et al. (208) have shown that the N6-methyladenosine (m6A) methyltransferase-like 3 (METTL3) is overexpressed in ESCC and is associated with a poor prognosis. METTL3 knockdown in ESCC cell lines TE9 and TE10 resulted in reduced proliferation, colony formation, and migration. m6A-methylated RNA immunoprecipitation-sequencing (MeRIP-seq) of ESCC cell lines revealed that METTL3-mediated m^6^A modifications regulate the notch signaling pathway, particularly Notch1 and its target gene, p21. Both METTL3 and NOTCH1 expression are positively correlated in ESCC. Next, METTL3 conditional knockout mice driven by the Keratin14 promoter were injected with the carcinogen 4-NQO, resulting in reduced esophageal lesions and tumor burden in METTL3 cKO mice compared to control mice. NOTCH1 and HES1 levels were reduced in METTL3 cKO tissues compared to control mice. Overexpression of METTL3 in conditional knock-in (74) mice resulted in enhanced 4-NQO-induced tumor growth compared to control mice. In contrast, increased expression of NOTCH1 and HES1 was observed in METTL3 cKI mice compared to control mice. These datasets suggested that METTL3 promoted m6A modifications, activated notch signaling, and promoted ESCC (208). Lysine-specific demethylase 1 (LSD1) is frequently deregulated in cancers. Hou and coworkers have recently shown the expression of LSD1 and Notch/PI3K pathway proteins in ESCC cell lines. Furthermore, the downregulation of LSD1 activity by TCP or shRNA resulted in decreased NOTCH1, 3, and HES1 levels in ESCC cells (ECa109 and EC9706), while chromatin immunoprecipitation (ChIP) analysis showed that the ability of LSD1 to bind to promoters of NOTCH2 and HES1 was also reduced after TCP treatment-induced inhibition of LSD1 in ECa109 cells. These datasets suggest that LSD1 regulates notch signaling in ESCC cells by binding to the promoters of notch pathway genes (209). Recently, long non-coding RNAs (lncRNAs) have shown great promise in cancer, including ESCC. Small Nucleolar RNA Host Gene 1 (SNHG1) is one such lncRNA that is overexpressed in ESCC, linked to metastasis, ESCC invasion, and staging, and associated with reduced overall survival. Moreover, SNHG1 knockdown in ESCC cells reduced proliferation and invasion and reversed EMT by enhancing E-cadherin expression and reducing vimentin and N-cadherin expression. SNHG1 knockdown inhibited the expression of notch signaling pathway proteins, including NOTCH1 and HES1, suggesting its role in regulating the notch signaling pathway in ESCC (210). The homeobox transcription factor MEIS1, which promotes ESCC progression, has been shown to be correlated with the notch signaling target gene Hey1 during the initial stages of ESCC development (211). Li et al. investigated oxidative stress and endoplasmic reticulum stress-related genes in EC using the TCGA and GEO databases, identifying TFRC as a potential biomarker for EC prognosis. TFRC expression was upregulated in EC, and its overexpression promoted the proliferation, migration, and invasion of EC cells (TE1 and OE33) by regulating the Notch1 signaling pathway (212). Chronic exposure to hexavalent chromium [Cr(VI)], a well-known carcinogen, causes malignant transformation [HEEC-Cr(VI)] in immortalized human esophageal epithelial cells (HEEC) by activating notch signaling. This pathway is crucial for maintaining cell proliferation and stem cell properties. HEEC-Cr(VI) cells show anchorage-independent growth, increased proliferation, CSC traits, and can form subcutaneous xenografts in BALB/c nude mice (213).

In summary, notch signaling in ESCC acts as both a tumor suppressor and a promoter, depending on the context, including genetic background, disease stage, and microenvironment. In normal esophageal tissue, notch signaling maintains epithelial homeostasis and differentiation, suppressing tumorigenesis. Its loss (e.g., NOTCH1 mutations, downregulation of NOTCH1, JAG1, JAG2, HES1) or inhibition (chemical carcinogens like 4-NQO, diethyl-nitrosamine) accelerates ESCC onset and increases tumor burden. During ESCC progression, notch signaling (especially NOTCH1 and NOTCH3) can switch to an oncogenic role, promoting epithelial-mesenchymal transition (EMT, involving SNAIL, ZEB1/2, TWIST1), stemness, metastasis, and therapy resistance. Notch contributes to drug resistance in ESCC: Enhanced NOTCH1 and NOTCH3 activity, upregulation of MAML1 (co-activator), and downstream targets (SOX2, SALL4, NANOG, HES1) are linked to resistance against chemotherapy and radiotherapy, partly by promoting cancer stem cell maintenance (CD44-high subpopulations) and EMT. The pathway’s effects are modulated by crosstalk with TGF-β (TGFB1), Wnt (CTNNB1/β-catenin), EGFR, MSI1, and metabolic/epigenetic regulators (PRMT1, POFUT1, METTL3, LSD1/KDM1A), as well as by non-coding RNAs (lncRNAs HAGLROS, SNHG1), inflammation (IL-23), and immune microenvironment factors. Notch pathway components (e.g., NOTCH1, NOTCH3, HES1, MAML1) serve as biomarkers for prognosis and therapeutic response, and as potential therapeutic targets—though clinical strategies require careful, context-dependent consideration given the pathway’s dual roles. This highlights the spectrum of notch signaling activities in ESCC, emphasizing its central but complex role in disease development, progression, and treatment. Collectively, notch signaling in ESCC operates on a spectrum: it suppresses tumor initiation by maintaining differentiation, but, upon genetic/epigenetic alterations or in specific microenvironmental contexts, it shifts toward tumor promotion—facilitating EMT, stemness, therapy resistance, and immune evasion. The pathway’s duality and extensive crosstalk position it as both a challenge and an opportunity for precision targeting in ESCC therapy (Table 5).

**TABLE 5:** Key roles of notch signaling in EAC.

Mechanisms	Description	Key genes/proteins involved
Initiation and metaplasia	Notch activation in DCLK1+ tuft cells at the squamocolumnar junction promotes Barrett’s esophagus (BE) formation through enhanced dysplasia and metaplasia.	NOTCH1, NOTCH2, DCLK1, HES1
Progression to EAC	Persistent NOTCH2/3 signaling suppresses goblet cell maturation and increases crypt fission, driving transition from BE to EAC, with decreased secretory differentiation.	NOTCH2, NOTCH3, HES1, MUC2
Crosstalk with inflammation and NF-κB	Notch interacts with inflammatory NF-κB signaling; acid reflux/inflammation upregulates DLL1, boosting notch and establishing a positive feedback loop for tumor progression.	DLL1, NF-κB (RelA/p65), NOTCH1, NOTCH2, NOTCH3 IL-6
Integration with TGF-β pathway	Loss of TGF-β repression enables notch to drive tumor-promoting genes (SOX9, c-MYC) and downregulate tumor suppressors, shifting TGF-β from suppressive to promoting.	SMAD4, β2-spectrin, NOTCH1, NOTCH2, SOX9, c-MYC, p21, p27, E-cadherin
CSC properties and redox regulation	Chronic reflux activates APE1-NF-κB, upregulating DLL1/Notch and promoting cancer stem cell traits, contributing to aggressiveness and therapy resistance.	APE1, NF-κB (RelA/p65), DLL1, NOTCH1, ALDH1, LGR5
Therapeutic implications	Targeting notch, NF-κB, DLL1, or APE1 may disrupt these circuits and offer strategies for EAC prevention and treatment.	NOTCH1-3, DLL1 NF-κB, APE1

#### 3.7.3 Notch signaling pathway in EAC

EAC begins in mucus-secreting cells of the esophagus and is most prevalent in the gastroesophagus. Obesity, GERD, and BE, a precancerous condition for EAC, are major risk factors. Notch signaling is implicated in BE and its progression to EAC, and several studies are underway to identify the mechanisms underlying this neoplastic progression (Table 5). Doublecortin-like kinase 1 (DCLK1) is upregulated in BE and EAC patients, particularly in epithelial and stromal regions (214). DCLK1 is also expressed in epithelial tuft cells at the SCJ and is proposed to be involved in esophageal tumor growth. Kunze et al. showed that notch expression in DCLK1-expressing tuft cells enhanced dysplasia, metaplasia, and BE development in *pL2—Dclk1-N2IC* mice (a mouse model of BE). Furthermore, deletion of Notch2 in DCLK1-expressing cells enhanced secretory cell differentiation and slowed BE progression in *pL2—Dclk1-N2IC* mice. Additionally, a microarray study was performed on Barrett’s whole tissue isolated from *pL2. Dclk1-N2IC* and *pL2. Dclk1-N2fl* mouse models to understand the transcriptomic landscape of BE progression. The analysis revealed increased expression of tumor-promoting pathways in BE tissues, suggesting a potential role for notch activation in DCLK1-expressing tuft cells in advancing BE (175). BE progression to EAC showed a characteristic reduction in goblet cell (GC) density compared to nondysplastic regions, while an increase in NOTCH3 and JAGGED2 mRNA levels was reported in tissues progressing from BE to EAC. Activated Notch2 (NICD2) in LGR5-positive cells decreased GC-like cell maturation, enhanced crypt fission, and promoted tumor formation in the SCJ area in mice, whereas Notch2 deletion from LGR5-positive cells enhanced GC-like cell maturation, decreased crypt fission, and reduced tumor development in mice. Furthermore, esophageal tissues isolated from *pL2-Lgr5-N2IC* mice showed enhanced RelA expression compared to L2-IL1B mice, with higher NF-κB activity in LGR5-positive cells, suggesting that notch signaling correlates with NF-κB activation. Deleting RelA from LGR5-positive cells reduced Notch2-induced tumor formation in pL2-Lgr5-p65fl/fl mice compared to *pL2-Lgr5-N2IC* mice. Furthermore, inhibiting NF-κB activation with JSH-23 reduced proliferation and survival in organoids derived from p*L2-IL1Beta* mouse tissues, suggesting a novel therapeutic strategy to prevent BE-to-EAC progression (215). Mendelson et al. studied the relationship between TGF-β and notch pathways in BE and EAC. They found reduced SMAD4 and β2-spectrin levels in patient tissues, with increased Hes1 and Jagged1 in EAC tissues and cells compared with normal tissues. Additionally, deficient TGF-β signaling, combined with activated notch signaling, is crucial for GSI-XXI to inhibit EAC cell proliferation (216). Loss of TGF-β adapter β2SP (SPNB2) was shown to activate notch signaling and SOX9 in EAC. shRNA knockdown of SPNB2 in SK-GT-4 cells increased SOX9, NICD1 nuclear translocation, spheroid formation, invasion, and tumor growth in mice. Reintroducing SPNB2 with the dnMAN mutant reduced SOX2 promoter activity. SPNB2 loss also enhanced SMAD3 and NICD1 binding, increasing SOX9 and c-MYC, while decreasing the expression of TGF-β target genes p21, p27, and E-cadherin. This indicates SPNB2 loss shifts TGF-β from tumor-suppressing to tumor-promoting by activating notch signaling (217). Chen et al. demonstrated increased mRNA levels of notch signaling components, including NOTCH1, NOTCH3, DLL1, DLL4, JAG1, and JAG2, as well as downstream targets *HES1* and *HEY1* in EAC. Furthermore, there was upregulation of NOTCH1, DLL1, DLL4, HES1, and HEY1 mRNA in EAC cell lines after reflux conditions (ABS exposure), while Western blot confirmed the upregulation of DLL1*,* including its cleaved form, and NICD protein after ABS exposure. Based on these datasets, the study identified novel crosstalk among reflux-associated inflammation, APE1-linked redox function, and increased DLL1 levels driven by NF-κB binding. The redox activity of the APE1 protein was important for activating NF-κB-Notch axes to induce CSC properties related to reflux conditions. Moreover, DLL1 and APE1 expression was high in human EAC tissues and L2-IL1ß mouse tissues, suggesting a novel role of DLL1 in acid reflux conditions in EAC (91).

In summary, notch signaling functions as a key regulator of EAC development and progression, coordinating environmental insults, chronic inflammation, and cellular differentiation. In EAC, notch activation occurs in specialized cell populations, including DCLK1-positive tuft cells and LGR5-positive stem-like cells at the squamocolumnar junction. Environmental factors such as chronic gastroesophageal reflux and bile exposure create an inflammatory microenvironment that triggers notch signaling, initiating BE—a key precursor to EAC. Experimental models show that notch activation in these lineages accelerates metaplasia and dysplasia, whereas notch inhibition restores secretory differentiation and slows BE progression. As BE advances to EAC, persistent notch activity—especially through NOTCH2 and NOTCH3—suppresses goblet cell maturation, increases crypt fission, and reduces secretory cell density, hallmark features of neoplastic transformation. Notch signaling intensifies crosstalk with proinflammatory pathways, particularly NF-κB, further promoting proliferation and tumorigenesis. This is evidenced by increased RelA expression and NF-κB activity in LGR5-positive cells, as well as by upregulation of notch ligands (such as DLL1) in response to inflammatory cytokines and acid reflux. A critical aspect of this network is the interplay between notch and TGF-β pathways. Loss of TGF-β pathway components (including SMAD4 and β2-spectrin) removes tumor-suppressive constraints on notch, enabling it to upregulate oncogenic drivers (SOX9, c-MYC) and downregulate tumor suppressors (p21, p27, E-cadherin). This molecular switch shifts TGF-β from a suppressive to a tumor-promoting role in EAC. Furthermore, chronic reflux conditions induce APE1-mediated redox activation of NF-κB and Notch, contributing to cancer stem cell phenotypes, tumor aggressiveness, and therapy resistance. Collectively, notch signaling serves as the nexus linking environmental, inflammatory, and molecular drivers of EAC. High expression of DLL1 and APE1 in human EAC tissues underscores their clinical relevance. Targeting notch and its interacting partners—NF-κB, DLL1, APE1, and components of the TGF-β pathway—represents a promising therapeutic strategy to disrupt the pathogenic circuitry underlying EAC (Table 5).

## 4 Targeting the notch signaling pathway using small-molecule inhibitors

**FIGURE 5:**
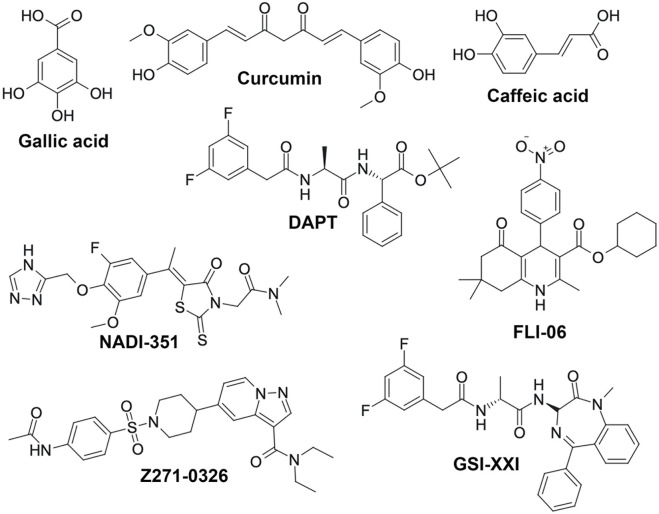
Chemical structures of notch inhibitors.

Inhibiting the notch pathway in EC is an emerging area of research (Figure 5). The following section summarizes studies targeting the notch pathway.

### 4.1 DAPT

Weaver et al. have identified NACK (Notch Activation-Complex Kinase) as a transcriptional co-activator of the Notch pathway. Both NACK and NOTCH1 were co-expressed, and NACK binding to the NICD complex was found to depend on MAML1. Moreover, NACK expression overlapped with Notch1 during embryonic development. Furthermore, shRNA-induced knockdown of NACK reduced colony formation in EAC cell lines OE19 and OE33 and inhibited tumor xenograft formation in OE19 cells. NACK knockout was embryonic-lethal in mice, underscoring the protein’s importance in development and cancer (218). Furthermore, NACK recruitment to the NOTCH1 ternary complex was mediated by p300-and CBP-mediated acetylation of MAML1 at *K188* and *K189*, which enables RNA polymerase II recruitment and induces transcription of notch signaling genes, such as HES1 and HEY1. Moreover, a ChIP assay with siRNA targeting p300 in the OE33 cell line showed that NACK localization on the HES1 promoter was mediated by p300. Furthermore, the combination of C646 (0.5 mg/kg) and GSI DAPT (0.5 mg/kg) inhibited p300 and had a synergistic effect on OE33 cell line growth *in vitro* and in the EAC47 patient-derived xenograft model (219). Recently, both the integrator complex, part of the NOTCH transcription complex, and NACK were required to activate notch signaling target genes, driving RNA polymerase II-dependent transcription and inducing EAC growth in cells and a mouse xenograft model. Furthermore, the integrator complex has been reported to be overexpressed in EAC cells, and knockdown of integrator subunit 11 has resulted in G2/M cell cycle arrest, programmed cell death, and inhibition of cell proliferation in EAC (220). Wang and colleagues have demonstrated that NICD is upregulated in EAC tissue samples compared with normal esophageal tissue using immunohistochemistry. Moreover, HES1, HEY1, HEY2, HEYL, JAG1, 3, DLL1, DLL3, and DLL4 mRNA levels were also higher in EAC tissues than in normal esophageal tissues. The activated notch signaling pathway has been associated with poor chemotherapeutic response in EAC patients. Furthermore, treatment with DAPT inhibited notch signaling, reduced the viability of OE33 and OE19 cells, and inhibited colony formation in OE33 and JH-EsoAdr1 cells. Moreover, shRNA-induced knockdown of CSL in the OE33 cell line also inhibited cell viability and colony formation. Furthermore, overexpression of NICD in Het1A cells increased colony formation, suggesting that notch activation-induced transformation occurred in Het1A cells. Finally, DAPT at 20 mg/kg, administered intraperitoneally, inhibited the growth of OE19 and OE33 tumor xenografts in mice. Similar effects were observed in patient-derived xenograft models (EAC28, EAC36, and EAC47) in NSG mice. Moreover, EAC CSC marker genes (*ALDH1A1, CD24, LGR5, SOX2, and TWIST1*) were sensitive to notch inhibition in spheroids treated with DAPT. Furthermore, treating EAC cell lines (FLO1, JH-EsoAd1, OE19, and OE33) with DAPT inhibited ALDH expression in 40%–60% of cells. Additionally, ALDH-expressing cells were more sensitive to DAPT than ALDH-negative cells. DAPT, in combination with 5-FU, suppressed the viability of OE33 cells at 1 and 5 μM concentrations. Overexpression of NICD in FLO1 cells induced 5-FU resistance. These datasets showed that notch induced chemotherapeutic resistance; hence, combination therapy with GSI has been proposed as a novel treatment option for EAC (221). Although several GSIs are currently undergoing Phase I-III clinical trials, including PF‐03084014 (Nirogacestat), LY3039478 (Crenigacestat), AL101 (Osugacestat), and LY900009, none are currently being investigated for use in esophageal cancer.

### 4.2 Z271-0326

A small-molecule inhibitor targeting NACK, designated Z271-0326, was recently identified through structure-based virtual screening and cell-based assays. The compound inhibited notch transcriptional activity by blocking NACK binding to the notch ternary complex. Furthermore, Z271-0326 inhibited the proliferation of OE19 (EC_50_ = 3.6 μM) and OE33 (EC_50_ = 3.14 μM) cells. Additionally, the compound inhibited NACK, NOTCH1, and MAML1 expression and reduced levels of notch target genes (*HES5, CCND1, and TBP*). Z271-0326 inhibited tumor spheroid formation compared with the control and showed greater efficacy than DAPT. Z271-0326 inhibited the growth of the OE19 tumor xenograft in mice without altering body weight. Moreover, the compound significantly inhibited the growth of patient-derived xenograft EAC47 (tumor volume and weight) at a dose of 15 mg/kg (220).

### 4.3 NADI351

In another study, NADI351 was developed as a NOTCH1 transcription complex inhibitor. The compound inhibited NOTCH transcriptional activity and HES1, a downstream target gene*.* NADI351 inhibited the proliferation of OE33 cells at an EC50 of 10 μM. Moreover, NADI351 (30 mg/kg, i. p.) inhibited the growth of patient-derived EAC47 and OE19 EAC xenografts in mice without affecting animal body weight. NADI351-treated tumors exhibited significant apoptosis (as indicated by TUNEL) and reduced ALDH1A and Ki-67 expression (a proliferation marker). Furthermore, NADI351 inhibited colony formation, HES1, HES5, and CCND1 expression, and spheroid formation in ALDH-expressing cells, but not in ALDH-deficient OE33 and EAC47 cells. Similarly, NADI351 was effective only in Notch1-positive OE33 cells, not in Notch1-negative cells. Moreover, NADI351 treatment reduced tumor growth in EAC47 xenografts in nude mice by decreasing NOTCH1-positive cells, suggesting a field-dependent notch mechanism (222). A novel histone demethylase GASC1 inhibitor, caffeic acid, and a GASC1-specific shRNA inhibited GASC1 levels in KYSE-150 and KYSE-30 ESCC cells. The data showed that caffeic acid and shRNA suppressed GASC1*,* NOTCH1, and MAPK pathways in the ESCC cell line (223).

### 4.4 FLI-06

FLI-06 was developed as a notch pathway inhibitor that disrupts the Golgi complex, thereby blocking pre-receptor secretion of the notch protein. FLI-06 treatment reduced the proliferation of ECa109 and EC9706 cells, with IC50 values of 5.81 and 10.74 μM, respectively, and induced apoptosis and G1 cell cycle arrest. Mechanistically, FLI-06 inhibited the expression of NOTCH3, HES1, DTX1, and LSD1. This LSD1 inhibition occurred in conjunction with DAPT-induced notch inhibition, suggesting that notch inhibitors control LSD1 expression while exerting antitumor activity (224).

### 4.5 EOAI3402143

Recently, the NOTCH1–USP5–STAT3 axis has been recognized as a key pro-angiogenic pathway in ESCC (225). The USP5 inhibitor EOAI3402143 (5 mg/kg, i. p.) was shown to block the effects of NICD1 and reduce tumor growth in mouse xenograft models using KYSE450 cells, including NICD1-overexpression xenografts. Additionally, EOAI3402143 demonstrated enhanced antitumor activity when combined with 5-FU/CDDP in NSG mouse ESCC xenograft models, in which mice were subcutaneously implanted with KYSE30 cells (225).

### 4.6 Anti-notch monoclonal antibodies

Brontictuzumab is a monoclonal antibody that binds to Notch1, thereby inhibiting Notch1 signaling. The initial human study aimed to determine the maximum tolerated dose (MTD), assess safety, pharmacokinetics, and immunogenicity, and evaluate the preliminary efficacy of Brontictuzumab in patients with solid tumors. The study included three patients with EC; however, no further research on Brontictuzumab in EC was reported (226). Another study described the development and evaluation of OMP-59R5 (taratumab) (227), a monoclonal antibody that blocks Notch2 and Notch3 signaling pathways, which are known to be critical in stem cell regulation and cancer. Overall, the findings support Notch2/3 blockade as a promising therapeutic strategy across multiple tumor types, but its effect on EC has not been studied. A bispecific antibody that targets VEGF and DLL4 (ABL001/NOV1501/TR009) shows enhanced biological activity both *in vitro* and *in vivo* compared to monoclonal antibodies targeting either VEGF or DLL4 alone. It is currently being tested in a Phase 1 clinical trial with cancer patients who have received heavy chemotherapy or targeted therapies. ABL001 showed synergistic antitumor efficacy against gastric and colon cancer xenografts when combined with paclitaxel and irinotecan in mice (228). Although a poster has been published regarding the dose-escalation study of NOV1501 in gastric cancer patients, which included some patients with gastro-esophageal cancer and demonstrated reduced tumor growth, the detailed findings of the study have not yet been published (229). Similarly, another bispecific antibody, HD10 (230), targeting VEGF and Dll4, more effectively inhibited tumor progression in human A549 lung and SCH gastric cancers than either an anti-VEGF antibody or an anti-Dll4 antibody alone. However, its impact on EC has not been studied, indicating a research gap on inhibiting notch signaling in EC in a clinical setting.

### 4.7 Naturally occurring polyphenols

Polyphenols are recognized for their ability to target CSCs and their associated signaling pathways within cancerous tissues (66). Consequently, several groups have summarized and examined the anticancer activity of various polyphenols and their analogs that have been researched for cancer treatment (59–66,231–243).

### 4.8 Curcumin

Curcumin is a polyphenolic compound isolated from turmeric that occurs naturally (243). Subramaniam et al. studied the effects of curcumin treatment on ESCC cells (TE-7, TE-10, ESO-1). Treatment inhibited cell growth and colony formation, induced apoptosis, and arrested the cell cycle by downregulating cyclin D1 *expression*. Furthermore, curcumin inhibited spheroid formation by reducing the size and number of primary and secondary spheroids in TE-7 and TE-10 cells, suggesting that curcumin affects CSC in ESCC. Curcumin treatment inhibited notch signaling pathway proteins NICD1, JAG1, and HES1 at both mRNA and protein levels. Mechanistically, curcumin has been shown to inhibit γ-secretase complex proteins, including PRESENILIN-1 and -2, NICASTRIN, APH1, and PEN2. Moreover, combining curcumin with GSI DAPT further inhibited cell growth and induced apoptosis by reducing the levels of HES1 and cyclin D1 (244).

### 4.9 Cranberry polyphenols

Weh et al. studied the effects of cranberry polyphenols on EAC initiation. Proanthocyanidin-enriched (C-PAC) extract and a combination extract of anthocyanins, flavonoids, and glycosides (AFG) were tested against BE and EAC cell lines. C-PAC exerted more potent inhibition of cell viability than AFG and induced apoptosis in EAC cell lines (JHAD1 and OE19). Furthermore, C-PAC treatment affected multiple proteins in EC and EAC cells, as demonstrated by reverse-phase protein arrays (RPPA). Notably, the notch signaling pathway (NOTCH1 and HES1) was significantly affected post-treatment, as observed in RPPA analysis. C-PAC treatment at 75 μg/mL in CP-B, CP-C, JHAD1, and OE19 cells potently inhibited the expression of cleaved Notch 1 and 2, as well as p-AKT (Ser473). More recent reports from the same group have revealed additional beneficial effects of C-PAC in EAC prevention and therapy, including suppression of reflux-induced DNA damage by upregulating *GSTT2*, mitigation of reflux-induced deregulation of transport proteins, and modulation of the esophageal metabolome (245–247). The data suggested the potential use of cranberry polyphenols to inhibit notch signaling in EAC (248).

### 4.10 Gallic acid

Recently, a plant-derived polyphenol, gallic acid, was shown to inhibit ESCC progression and sensitize ESCC cells to cisplatin. Gallic acid inhibited proliferation and induced apoptosis in KYSE30 and TE-1 ESCC cell lines. Furthermore, gallic acid treatment inhibited IL-6-induced activation of the STAT3 and notch pathways and reduced proliferation, colony formation, migration, and invasion in ESCC cells (KYSE30 and TE-1). In contrast, inhibitors of STAT3 (S31-201) or Notch (GSI:DAPT) potentiated the effects of gallic acid. Finally, gallic acid induced oxidative stress and increased the sensitivity of ESCC cells to cisplatin, both *in vitro* and *in vivo*, in a tumor xenograft model using the cisplatin-resistant TE-1 cell line in nude mice (249).

### 4.11 Caffeic acid

Caffeic acid, and shRNA specific to GASC1 inhibited GASC1 levels in KYSE-150 and KYSE-30 ESCC cells. The data showed that Caffeic acid and shRNA suppressed the GASC1, NOTCH1, and MAPK pathways in the ESCC cell line (223).

## 5 Conclusion and summary

EC is a significant problem worldwide, while EAC is a major problem in Western countries, including the US and European countries. The current chemotherapy regimen did not differentiate extensively between EAC and ESCC. The CROSS trial (250) showed that overall survival and pathological response were significantly better in ESCC patients compared to EAC with neoadjuvant chemoradiotherapy (nCRT) followed by surgery *versus* surgery alone for patients with resectable esophageal or esophagogastric junctional cancer. Hence, there is a need to develop therapies that improve survival in EAC patients. Moreover, current chemotherapy is insufficient to eliminate EC CSCs, and thus, EC tumors exhibit aggressiveness and chemoresistance. The presence of these CSCs represents a population of pluripotent, invasive, and treatment-resistant cells and may explain why chemoradiotherapy for these tumors is sometimes inadequate. The addition of CSC-targeting drugs to current chemotherapy may further improve locoregional disease control, reduce the risk of distant metastases, and enhance overall survival in EC. Attempts to discover agents targeting CSCs have involved targeting surface markers (ALDH1A1, LGR5, SOX2, TWIST1), inhibiting their signaling pathways, or modifying their microenvironment to make them more vulnerable to treatment. While several signaling pathways are involved in EC CSCs, the notch signaling pathway has shown promise in EC tumor progression and drug resistance. Attempts have been reported to develop inhibitors of the notch signaling pathway, especially GSIs. Despite strong preclinical evidence supporting notch pathway inhibition in EC, clinical translation has been limited. Early studies with GSI revealed dose-limited gastrointestinal toxicity due to on-target disruption of epithelial homeostasis. These challenges highlight the need for optimized dosing strategies such as intermittent schedules, combination therapies that permit dose reduction, and the development of more selective agents. Patient stratification is critical for the clinical efficacy of notch-targeted therapies, as notch signaling exhibits context-dependent function across EAC and ESCC and varies by disease stage; hence, there is a need for biomarker-guided stratification. Clinical benefit is likely restricted to tumors with active notch signaling or enrichment of CSC-like population; therefore, molecular profiling should be integrated into clinical trial design. There is a need to develop specific notch inhibitors in combination with current chemotherapy in clinical settings to improve chemotherapy efficacy, pathological response, and overall survival in EC patients.
